# Indonesian Dental Students' Attitudes, Knowledge, Preparation, and Willingness to Treat HIV/AIDS Patients

**DOI:** 10.1055/s-0041-1740350

**Published:** 2022-01-06

**Authors:** Yuniardini Septorini Wimardhani, Yuli Fatzia Ossa, Indriasti Indah Wardhany, Diah Ayu Maharani, Cliff Lee

**Affiliations:** 1 Department of Oral Medicine, Faculty of Dentistry, Universitas Indonesia, Jakarta, Indonesia; 2 Oral Medicine Residency Program, Faculty of Dentistry, Universitas Indonesia, Jakarta, Indonesia; 3 Department of Preventive and Public Health Dentistry, Faculty of Dentistry, Universitas Indonesia, Jakarta, Indonesia; 4 Department of Oral Medicine, Immunity and Infection, Harvard School of Dental Medicine, Boston, Massachusetts, United States

**Keywords:** HIV, dental care, attitude, willingness to treat, dental education, Indonesia

## Abstract

**Objective** To assess the Indonesian dental students' knowledge of HIV/AIDS in terms of transmission and oral manifestation, the attitudes toward people living with HIV/AIDS (PLWHA), the preparedness in infection control, and willingness to treat PLWHA, and assess the factors for willingness to treat PLWHA.

**Materials and Methods** A modified version of a questionnaire used to assess dental students' knowledge, attitude, preparedness, and willingness to treat PLWHA in China was used. The questionnaire was cross-culturally adapted into Indonesian and had been pre-tested for face validity and test and retest reliability. The dental students from 32 dental schools in Indonesia were invited to participate in the study.

**Results** A total of 1,280 dental students from 23 dental schools participated in the study. This study found that only 63% of students scored higher than 70% for knowledge of HIV/AIDS, and the mean score for knowledge was 15.02 (2.4). Higher than 80% of students had a positive professional attitude toward PWLHA; however, 80% of students worried about possible disease transmission in the dental office by PLWHA and 70% of students overestimated the occupational risk when treating PLWHA. The dental students had good preparedness for infection control with a mean score for preparedness of 3.19 (0.4). The mean score for willingness was 2.5 (0.9). Willingness to treat significantly differed by the type of universities, gender, age, and clinical experience. This study showed that knowledge about HIV/AIDS correlated with the willingness to treat PLWHA among dental students.

**Conclusion** Dental students who have good knowledge about HIV infection tend to have a good willingness to treat PLWHA. The knowledge would in turn affect their attitude, preparedness, and willingness to provide care for PLWHA with confidence and comfort. This study suggests that the improvement of dental training may play an important role in changing students' perception of willingness to treat PLWHA.

## Introduction

In Indonesia, as of December 2020, an estimated 543.100 people was living with HIV/AIDS (PLWHA) which is ∼0.2% of the total population of Indonesia. This number has continued to grow since first reported in 1987. 1 There are some oral manifestations associated with HIV or with its treatment that are related to the progress of HIV infection. 2 These oral manifestations may cause pain and dysfunction in the mouth. When the condition is left untreated, it may influence the systemic condition and quality of life for PLWHA. 3 However, many studies have shown that PLWHA faces barriers when seeking care. Stigma from healthcare workers is one of the identified barriers for PLWHA to having dental care they need. 4 Some factors related to barriers from dental providers include fear of HIV transmission, disease-related, and social stigma, and the limited knowledge about the oral manifestation of HIV. 5 6 Data showing the unmet dental care needs of PLWHA in Indonesia have not been identified; however, in the USA, the proportion of PLWHA with unmet dental care reached up to 65%. 7 8

Physicians and dentists are expected to properly treat PLHWA as stated by the World Health Organization (WHO) over 30 years ago on the first World's AIDS day in 1988. However, even now, negative attitudes and refusal to treat PLWHA are still documented in many countries worldwide. 9 In Indonesia, there has not been a national survey to explore dentists' knowledge, attitude, and willingness to treat PLWHA; however, previous studies in several cities and institutions in Indonesia on the topic have been done. 10 11 12 Overall, the results indicated the need to improve the willingness to treat PLWHA although the results were contradictory in a study of a single institutional study. 12 An increase in the number of PLWHA seeking dental care is expected as the population continues to grow. To be able to provide optimal care with confidence and comfort, dentists should be equipped with proper knowledge about HIV/AIDS, a positive attitude, and adequate infection control. Dentists should have continuing education related to the topic and dental students as future dentists should have education and training that could cover those aspects. 13

The eighth World Workshop on Oral Health and Disease in AIDS (WW8 AIDS) held in Bali 2019 issued a declaration for ending the stigma against HIV/AIDS. 14 The contents of the declaration included the role of the dentist recognized as an integral part of the healthcare team committed to achieving the aims of the United Nations Programme on HIV/AIDS (UNAIDS). The HIV education of dental students and dental healthcare workers should be an essential element of the dental curriculum to ensure the profession has the appropriate knowledge and attitudes to manage PLWHA, and public health programs make certain that the general public is aware of the role of dental healthcare workers and normalizing attitudes to PLWHA. 14 Previous studies in several countries such as China, Malaysia, India, the UK, Egypt, and Africa have shown that there was still a need for improvement in shaping dental students' attitudes to prepare them to treat PLWHA by increasing their knowledge. 9 15 16 17 18 19 20 Currently, studies that explore the students' knowledge, attitude, preparedness, and willingness to treat PLWHA are still limited in Indonesia; therefore, a comparison of results to those previous studies could not be performed. As knowledge, attitude, and preparedness in infection control are some factors that were indicated to influence the willingness to treat PLWHA, this study aimed to assess the Indonesian dental students' knowledge of HIV/AIDS in terms of transmission and oral manifestation, the attitudes toward PLWHA, the preparedness in infection control, and the willingness to treat PLWHA. In addition, the relationship between these factors and willingness to treat PLWHA was also explored.

## Materials and Methods

### Study Design

This was a cross-sectional questionnaire-based study to assess the knowledge, attitude, preparedness, and willingness of dental students across Indonesia to treat patients with HIV/AIDS. This study was approved by the Research Ethics Committee of the Faculty of Dentistry Universitas Indonesia (No. 67/Ethical Approval/FKGUI/VIII/2019). The questions used were modified from a questionnaire developed and reported in a previous study. 16

### Questionnaire

The questionnaire was cross-culturally adapted using the guidelines for cross-cultural adaptation. All the cross-adaptation process was performed to finally use the Indonesian version of the questionnaire. 21 22 The questionnaire consisted of five sections, which were demographic data, knowledge (21 questions, scale 0–1), attitude (14 questions, scale1–4), preparedness (16 questions, scale 1–4), and willingness (1 question, scale 1–4). In the knowledge section, students were asked to provide a “true” or “false” or “do not know.” Each right answer was scored 1, and false or do not know the answer was scored 0. For attitude, preparedness, and willingness, a 4-point Likert scale was used. The data analysis was based on the total score value of the answers for knowledge, attitude, preparedness, and willingness analyzed based on the mean Likert score.

### Study Participants

The sampling frame consisted of all students from 32 dental schools in Indonesia who were at the clinical stage of the dentistry program (years 4 and 5). Permission from all Deans of the dental schools was granted before the distribution of the questionnaire. The questions were arranged in an electronic form (Google Form) and distributed to the students through each dental school coordinator. After the permission was granted from each school, the questionnaire was distributed to the students and opened for 2 weeks. The students provided written informed consent to participate in the study. Any participant who did not complete the questionnaire was excluded from the study. Data were collected during October–November 2019.

### Data Analysis

The statistical analyses were performed using SPSS software version 23.0. Descriptive statistics were calculated, and bivariate analyses were performed. The relationships between the levels of knowledge, attitude, preparedness, and willingness to treat PLWHA with sociodemographic characteristics were analyzed using Mann–Whitney and Kruskal–Wallis tests. Correlation of knowledge, attitude, and preparedness with the willingness to treat PLWHA was done using Spearman's correlation analysis. A significance level of 0.05 was used for all analyses.

## Results

The demographic data of respondents are shown in Table 1 . There were 23 (71.9%) dental schools that agreed to participate in the study. The total number of respondents was 1,280 with the number of female students five times higher than male students. The number of students who participated from public universities was almost 10% higher than those from private ones. Most of the students were in their second year of the clinical year (49.5%).

**Table 1:** Characteristics of students who participated to the study

Characteristics	*n* (%)
Type of university	
Public	701 (54.8)
Private	579 (45.2)
Sex	
Male	212 (16.5)
Female	1068 (83.3)
Age	
< 25	1015 (79.3)
≥25	265 (20.7)
Year of clinical experience	
1 year	370 (28.9)
2 years	634 (49.5)
> 2 years	276 (21.6)

### Knowledge about HIV

Overall, 63% of students scored 70% or higher. Almost all students correctly answered blood as a mode of transmission; however, only 11% of students could recognize that HIV could be potentially transmissible through aerosols ( Table 2 ). Furthermore, 65% of students thought that sputum could transmit HIV, and 50.9% of students incorrectly believed that saliva was a way of HIV transmission. Around 25% of students thought that HIV is transmissible through insect bites and tears and only 20% of students were aware of the number of PLWHA in their region. In terms of knowledge of oral manifestations, the lowest percentage of correct answers from students was about xerostomia and aphthous ulcers. Many students mistakenly believe that oral lichen planus and peripheral ossifying fibroma were oral manifestations of HIV. Students from public universities had a higher score of knowledge than those from private universities and the difference was statistically significant ( *p* < 0.05) ( Table 5 ).

**Table 2:** Indonesian dental students' knowledge of HIV/AIDS

Knowledge		Responses ( *N* = 1280)			
		Correct		Incorrect	
		*n*	%	*n*	%
Mode of HIV transmission					
Yes	Aerosol (contaminated by blood)	142	11.1	1138	88.9
	Blood	1273	99.5	7	0.5
	Breast milk	1080	84.4	200	15.6
	Semen	1139	89.0	141	11.0
	Vaginal secretion	1164	90.9	48	3.8
No	Insect bites	932	72.8	348	27.2
	Sputum	438	34.2	842	65.8
	Saliva	628	49.1	652	50.9
	Tears	961	75.1	319	24.9
Oral manifestations related to HIV/AIDS					
Yes	Kaposi's sarcoma	742	58.0	538	42.0
	Oral candidiasis	1177	92.0	103	8.0
	Acute necrotizing ulcerative gingivitis	876	68.4	404	31.6
	Hairy leukoplakia	1022	79.8	258	20.2
	Herpes simplex lesion	779	60.9	501	39.1
	Xerostomia	627	49.0	653	51.0
	Aphthous ulcer	648	50.6	632	49.4
No	Fordyce granules	900	70.3	380	29.7
	Peripheral ossifying fibroma	593	46.3	687	53.7
	Lichen planus	440	34.4	840	65.6
Blood has the highest virus concentration		1124	87.8	156	12.2
Knowledge about number of PLWHA in the region		269	21.0	1011	79.0

### Attitude Toward PLWHA

Table 3 shows the Indonesian dental students' attitudes toward PLWHA. This study used 14 statements to assess students' attitudes toward PLWHA. The statements were divided into three different domains. The domains were professionalism, opinion and feelings, and personal assurance. Higher than 80% of students had a positive professional attitude toward PLWHA. In the opinions and feeling domain, the majority felt that their professional education had provided enough information to work safely with PLWHA and they agreed that they should not refuse to treat PLWHA and did not have negative feelings toward PLWHA. However, ∼80% of the students still had worries that PLWHA were responsible to transmit the disease and would refer the patient to be treated elsewhere. In the personal assurance domain, only almost 50% of students were sure that they knew the post-exposure procedures/protocols, and 70% of students were worried about their safety when treating PLWHA. Overall, the results showed that dental students tended to have a positive attitude toward PLWHA with a mean score of 3.04 (0.3). None of the students' characteristics were associated with the attitude toward PLWHA ( Table 5 ).

**Table 3:** Indonesian dental students' attitude toward PLWHA

Domain	Strongly disagree	Disagree	Agree	Strongly agree
	1 *n* (%)	2 *n* (%)	3 *n* (%)	4 *n* (%)
1. Professionalism				
You need more training/learning about HIV oral manifestations and how to treat PLWHA in the faculty of dentistry	17 (1.3)	31 (2.4)	243 (19.0)	989 (77.3)
HIV status must be disclosed and become part of the patient's archive	47 (3.7)	86 (6.7)	328 (25.6)	819 (64.0)
You have ethical responsibilities to provide dental care to PLWHA	24 (1.9)	83 (6.5)	430 (33.6)	743 (58.0)
All patients must be considered potentially infectious	58 (4.5)	85 (6.6)	339 (25.5)	798 (62.3)
You must behave toward PLWHA as well as non-HIV patients	78 (6.1)	134 (10.5)	421 (32.9)	647 (50.5)
2. Opinion and feelings				
If you have an option, you would prefer to refer HIV patient to anywhere else	80 (6.3)	169 (13.2)	528 (41.3)	520 (40.6)
Professional health workers should not refuse to treat PLWHA	174 (13.6)	294 (23.0)	446 (34.8)	366 (28.6)
You do not have negative feelings toward people living with HIV	111 (8.7)	307 (24.0)	501 (39.1)	361 (29.2)
Your professional education has provided enough information to work safely with PLWHA	26 (2.0)	101 (7.9)	425 (35.3)	701 (54.8)
You think your patients will be worried of they know you have treated PLWHA	63 (4.9)	169 (13.2)	528 (41.3)	520 (40.6)
PLWHA are responsible for transmitting the disease	98 (7.7)	261 (20.4)	420 (32.8)	501 (39.1)
3. Personal assurance				
You know the HIV post-exposure procedures/protocols	235 (18.4)	453 (35.4)	432 (33.0)	169 (13.2)
You are worried (or may worry) about your own safety when treating PLWHA	77 (6.0)	295 (23.0)	513 (40.1)	395 (30.9)
Your view of people living with HIV does not change the way you treat PLWHA	121 (9.5)	261 (20.4)	544 (42.5)	354 (27.7)

### Preparedness of Infection Control

Table 4 shows the Indonesian dental students' preparedness regarding infection control when treated PLWHA. Although they agreed that all patients should be considered potentially infectious, the majority of the students would change/enhance their practice of infection control when treating known PLWHA. There was still a low percentage of dental students who were not prepared to practice proper infection control. For example, there were 3.0% of dental students who rarely or only sometimes routinely wore gloves when doing procedures. The results of this study showed that overall, dental students had good preparedness for infection control with a mean score for preparedness of 3.19 (0.4). Students who were female and from the public universities had a higher preparedness score than those who were male and from the private universities ( *p* < 0.05) ( Table 5 ).

**Table 4:** Distribution of dental students preparations regarding infection control when treated for PLWHA

Domain	Rarely	Sometimes	Usually	Always
	1 *n* (%)	2 *n* (%)	3 *n* (%)	4 *n* (%)
Infection control practice				
Wearing gloves during the procedure	12 (0.9)	27 (2.1)	394 (30.8)	847 (66.2)
Changing glove after patient's treatment	16 (1.3)	26 (2.0)	377 (29.5)	861 (67.3)
Sterilize the instrument before use	15 (1.2)	30 (2.3)	387 (30.2)	848 (66.3)
Changing instruments between patients	29 (2.3)	44 (3.4)	383 (29.9)	824 (64.4)
Wearing personal protective equipment (excluding gloves)	40 (3.1)	109 (8.5)	441 (34.5)	690 (53.9)
Retrieve/update the patient's medical history	39 (3.0)	132 (10.3)	491 (38.4)	618 (48.3)
Using disinfectants and /or replacing protectors on the unit	41 (3.2)	147 (11.5)	481 (37.6)	611 (47.7)
Sterilizing personal protective equipment	55 (4.3)	187 (14.6)	488 (38.1)	550 (43.0)
Appropriate handling of patient care				
Change treatment to examine specific HIV manifestations	130 (10.2)	196 (15.3)	522 (40.8)	432 (33.8)
Extra or different sterilization/disinfection	42 (3.3)	66 (5.2)	530 (41.4)	642 (50.2)
Refer to a public health clinic	149 (11.6)	245 (19.1)	425 (35.3)	434 (33.9)
Change treatment to shorten procedures	378 (29.5)	347 (27.1)	335 (26.2)	220 (17.2)
Treat in different room/location	278 (21.7)	301 (23.5)	372 (29.1)	329 (25.7)
Wearing personal protective equipment	34 (2.7)	68 (5.3)	529 (41.3)	649 (50.7)
Inform your staff about the patient's health status	122 (9.5)	127 (9.9)	452 (35.3)	579 (45.2)
Request the patients to use protective goggles	395 (30.9)	427 (33.4)	261 (20.4)	197 (15.4)

**Table 5:** Distribution of students based on their knowledge, attitude, preparedness, and willingness related to HIV/AIDS

Characteristics	Knowledge of HIV		Attitude toward PLWHA		Preparedness in infection control		Willingness to treat PLWHA	
	Mean (SD)	*p* -value	Mean (SD)	*P* -value	Mean (SD)	*P* -value	Mean (SD)	*P* -value
Type of university								
Public	15.52 (2.4)	0.001 *	3.04 (0.3)	0.33	3.19 (0.4)	0.009 *	2.59 (0.9)	0.037 *
Private	14.52 (2.5)		3.05 (0.3)		3.12 (0.4)		2.49 (0.9)	
Sex								
Male	14.75 (2.5)	0.12	3.03 (0.3)	0.73	3.07 (0.5)	0.01 *	2.38 (0.8)	0.08 *
Female	15.13 (2.5)		3.05 (0.3)		3.17 (0.4)		2.57 (0.9)	
Age								
<25	15.0 (2.5)	0.25	3.04 (0.3)	0.32	3.17 (0.4)	0.11	2.57 (0.9)	0.01 *
≥25	15.2 (2.5)		3.06 (0.4)		3.11 (0.5)		2.40 (0.9)	
Year of clinical experience								
1 year	14.87 (2.6)	0.20	3.02 (0.3)	0.44	3.18 (0.4)	0.34	2.51 (0.9)	0.03**
2 years	15.19 (2.3)		3.05 (0.3)		3.17 (0.4)		2.60 (0.9)	
> 2 years	15.06 (2.6)		3.01 (0.4)		3.10 (0.5)		2.43 (0.9)	

* *p* < 0.05 Mann–Whitney test*; and Kruskal–Wallis test**.

### Willingness to Treat PLWHA

Dental students' willingness to treat PLWHA had the lowest score compared with other aspects that were explored in this study. The dental students were asked about their willingness to treat PLWHA. Only 14.5% of dental students were willing to treat without any doubt. The mean score for willingness was 2.5 (0.9). Table 5 describes the distribution of the willingness mean score by students' characteristics. The score was significantly differed by the type of university, gender, age, and clinical experience.

### Correlation of Knowledge, Attitude, and Preparedness with Willingness to Treat PLWHA

This study did not find any correlation between attitude and preparedness of infection control with students' willingness to treat PLWHA ( *r* < 0.1). Although the correlation was very low between knowledge of HIV/AIDS with the willingness to treat, the correlation was statistically significant ( *p* < 0.05). The knowledge of HIV/AIDS and attitude toward PLWHA also had a statistically significant correlation with the preparedness for infection control ( *p* < 0.05). Table 6 describes the *r* -value of Spearman's correlation analysis of variables.

**Table 6:** Correlations (Spearman's correlations) between knowledge, attitude, preparedness, and willingness related to HIV/AIDS among dental students in Indonesia

	Knowledge	Attitude	Preparedness	Willingness
Knowledge	1			
Attitude	0.01	1		
Preparedness	0.08 *	0.20 *	1	
Willingness	0.06 *	0.01	0.01	1

* Correlations were significant at *p* < 0.05.

## Discussion

This study investigated several aspects related to knowledge, attitude, preparedness, and willingness of Indonesian dental students toward PLWHA. This study aimed to explore the relation of dental students' knowledge, attitude, and preparedness with the willingness to treat PLWHA. This study was performed as a response to the trend of HIV infection worldwide and in Indonesia and attributed to the recent publication related to HIV education that suggested that there is a need to evaluate the course format and content to appropriately provide education and training to dental students. 23 Students from more than half of the 32 dental schools in Indonesia participated in this study, with a representative composition in relation to age, clinical experience, and type of university. However, females seemed to be overrepresented and response bias may not be ruled out. We adapted the previously used questionnaire to assess the same aspects in Indonesian settings. This study used a questionnaire that was applied for research in China. 16 The original questionnaire was in English and Chinese that was cross-culturally adapted into Indonesian using the previously published method. 21

The knowledge about HIV/AIDS correlated with willingness to treat PLWHA among Indonesian dental students. Many studies have indicated the need for more interactive activities that stimulate experiential learning for dental students on HIV/AIDS topics that may influence their knowledge gain, empathy, and reduce stigma; therefore, attributed to the increase of willingness to treat PLWHA. 23 Fear and stigma related to PLWHA is a problem faced by many countries and not only confined to countries with a specific culture or religion. 9 Studies have shown that the unwillingness to treat PLWHA resulted from preconceived assumptions and misconceptions about the condition. 24 25 Other factors also include the fear of being infected with HIV, personal values, religious and socio-cultural values, and norms. 26 27 A recent study in Indonesia showed that stigma among health care providers toward PLWHA is still high. 26 Based on the results of this study, improvement of the dental curriculum on HIV/AIDS may significantly improve attitudes toward and willingness to treat PLWHA. 28 Several studies have argued that the methods of learning related to HIV/AIDS topics should be modified during dental training. Besides emphasizing teaching, the knowledge about HIV/AIDS transmission, universal precaution, teaching appropriate technical skills, and interpersonal skills need to deliver effective dental care to PLWHA is also important. As also been proposed by other studies, opportunities to have an interaction with PLWHA would be essential to ensure dental students to be professional and competent in managing PLWHA in Indonesia. 16 23

The Indonesian dental students tended to have a positive attitude toward PLWHA, and none of the students' characteristics were associated with the attitude toward PLWHA. The dental students' attitude had a correlation with their preparedness related to infection control. Although in general, dental students had a positive attitude toward PLWHA, the overwhelming majority would prefer to refer PLWHA elsewhere if given the choice. These results were similar to previous studies in China, Kuwait, Brazil, and Canada. 16 29 30 31 The students overestimated disease transmission by PLWHA in dental practice, which made the negative attitude of thinking that they should be treated somewhere else. This worry may be attributed to the misperception of the risk of HIV transmission in dental practice as a result of occupational risks. A previous study showed that oral health care workers did not know that saliva contains anti-HIV activity and erroneously believed that saliva without blood contamination could transmit HIV, and thought that dental professionals have a high risk of being infected by HIV compared with other health care providers. 32 The students were still worried about their safety when treating PLWHA despite practicing universal precaution, which can translate to negative attitudes toward PLWHA. 33 Dental training includes dental professionalism that emphasizes equal and non-judgmental service to all; however, many studies showed that many biases that influence professionalism are experienced by PLWHA. 4 34

The majority of students practiced proper infection control when treating patients with standard/universal precaution. 35 The students also agreed that they should treat all patients to be potentially infectious. This study showed that female students and students from public universities significantly had a higher score of preparedness for infection control. Although a previous study believed that the gender difference did not influence the access to oral care to PLWHA, the possible explanation for this results may be because males were actually more concerned about the cost with regard to spending for infection control compared with females. 30 This study showed that only a small proportion of dental students knew the range of the number of PLWHA in their province, so we could not see the relation whether this could cause more compliance with infection control, as seen in the previous study. 16 Dental students in this study answered that they would be more cautious when they know they are treating PLWHA and make extra protection in terms of the PPE and patient wearing goggles, which is in contradiction to universal precaution. The infection control measures in the healthcare setting for universal precaution is for bloodborne pathogens such as HIV transmission, and not the same standard for respiratory transmission such as with the SARS-CoV-2. 36 37 The data collection for this study was performed before the pandemic started.

Only 50% of dental students had a positive answer that they will treat PLWHA without any doubt. This study showed that dental students' willingness to treat PLWHA without any doubt significantly differed by the type of university, gender, age, and year of clinical experience. The results were similar to other previous studies that showed this pattern. 10 30 38 The students have had 1 to 2 years' clinical activities in the dental schools at the time the study was conducted. This study showed that the long experience in clinical activities had improved scores in the knowledge about HIV/AIDS as well as the willingness to treat PLWHA. In our study, the majority of respondents already knew correctly the mode of HIV/AIDS transmission, which is in line with the research on the transmission of HIV disease that was conducted in Israel. 38 However, in this study, there were still some dental students who did not have correct answers about the transmission mode of HIV. This lack of knowledge may reflect that there is still room for improvement to increase the students' knowledge. Overall, this study showed that students from public dental schools had a higher score compared with the private dental school's ones in terms of knowledge, preparedness, and willingness to treat PLWHA. The results of this current study are completely comparable with those of a previous study in China. 16 These results implicate a possible room for improvement related to the increase in the knowledge in dental students, implemented in the same standard of learning method related to the subject throughout dental schools in the country. The previous study in India has indicated that training and education in an advanced curriculum provided to dental students were sufficient to handle PLWHA. 13

## Conclusion

Several aspects related to knowledge, attitude, preparedness, and willingness of Indonesian dental students toward PLWHA were explored. This study demonstrated the need to improve teaching methods to increase the knowledge of dental students, implemented in the same standard of learning method related to the subject throughout dental schools in the country. The methods that would stimulate experiential learning for dental students on HIV/AIDS topics that may influence their knowledge gain, empathy, and reducing stigma; therefore, attributed to the increase in the willingness to treat PLWHA. The role of dental schools in Indonesia should be to provide dental training, specifically implementing modifications in the methods of learning for HIV/AIDS, which would be essential to increase oral care access for PLWHA in the future.

## References

[1] Indonesian HIV/AIDS information system[Internet] Accessed July 1, 2021 at:https://siha.kemkes.go.id/portal/files_upload/Laporan_TW_IV_2020.pdf

[2] Patton L L Oral lesions associated with human immunodeficiency virus disease Dent Clin North Am 2013 57 04 673 698 24034072 10.1016/j.cden.2013.07.005

[3] Patton L L Phelan J A Ramos-Gomez F J Nittayananta W Shiboski C H Mbuguye T L Prevalence and classification of HIV-associated oral lesions Oral Dis 2002 8 02 98 109 12164670 10.1034/j.1601-0825.2002.00020.x

[4] Brondani M A Phillips J C Kerston R P Moniri N R Stigma around HIV in dental care: Patients' experiences J Can Dent Assoc 2016 82 g1 27548661

[5] Brown L Macintyre K Trujillo L Interventions to reduce HIV/AIDS stigma: what have we learned? AIDS Educ Prev 2003 15 01 49 69 12627743 10.1521/aeap.15.1.49.23844

[6] Wagner A C Hart T A McShane K E Margolese S Girard T A Health care provider attitudes and beliefs about people living with HIV: Initial validation of the Health Care Provider HIV/AIDS Stigma Scale (HPASS) AIDS Behav 2014 18 12 2397 2408 24965675 10.1007/s10461-014-0834-8

[7] Jeanty Y Cardenas G Fox J E Correlates of unmet dental care need among HIV-positive people since being diagnosed with HIV Public Health Rep 2012 127 02 17 24 22547873 10.1177/00333549121270S204 PMC3314389

[8] Fox J E Tobias C R Bachman S S Reznik D A Rajabiun S Verdecias N Increasing access to oral health care for people living with HIV/AIDS in the U.S.: baseline evaluation results of the innovations in Oral Health Care Initiative Public Health Rep 2012 127 02 5 16 22547872 10.1177/00333549121270S203 PMC3314388

[9] Abou El Fadl R K Abdelmoety A Farahat Z Hussein M A Assessing the levels of HIV-related knowledge and attitudes toward HIV-infected patients among undergraduate dental students: a cross-sectional study HIV AIDS (Auckl) 2019 11 83 92 31114391 10.2147/HIV.S195984 PMC6485033

[10] Gunardi I Salsabila Nurina N Marcia M Amtha R Dentists experience influences knowledge and attitudes toward HIV patients in West Jakarta, Indonesia, and validation of a new questionnaire Oral Dis 2020 26 01 127 132 32862523 10.1111/odi.13393

[11] Kadrianto T H Subita G P Aliyah S Willingness of dentists in Jakarta in treating patients with HIV/AIDS. Thesis Universitas Indonesia 2014

[12] Pratiwi R Akbar F H Pasiga B Description of the level of knowledge, preparedness and willingness of the Faculty of Dentistry, Hasanuddin University in caring for people with HIV/AIDS J Int Dent Med Res 2019 12 02 688 694

[13] Vijayalaxmi N Reddy L Swapna L A Reddy S Ramesh T Padmareddy M Are you willing to treat patients with HIV/AIDS? An anonymous survey among staff and students of dental institution Oral Health Dent Manag 2014 13 03 745 748 25284550

[14] FDI World Dental Federation The Bali Declaration on Oral Health in HIV/AIDS 2019. Published 2019. Accessed December 9, 2019 at:https://www.fdiworlddental.org/bali-declaration-oral-health-hivaids-dentists-can-help-end-epidemic-2030

[15] Singh V P Osman I S Rahmat N A Bakar N AA Razak N FNA Nettem S Knowledge and attitude of dental students towards HIV/AIDS patients in Melaka Malays J Med Sci 2017 24 03 73 82 10.21315/mjms2017.24.3.9 PMC5545620 28814935

[16] Lee C Fan Y Starr J R Dogon I L Dentists' and dental students' attitudes, knowledge, preparedness, and willingness related to treatment of people living with HIV/AIDS in China J Public Health Dent 2017 77 01 30 38 27427861 10.1111/jphd.12168

[17] Azodo C C Ehigiator O Oboro H O Nigerian dental students' willingness to treat HIV-positive patients J Dent Educ 2010 74 04 446 452 20388818

[18] Seacat J D Litt M D Daniels A S Dental students treating patients living with HIV/AIDS: the influence of attitudes and HIV knowledge J Dent Educ 2009 73 04 437 444 19339430

[19] Fotedar S Sharma K R Sogi G M Fotedar V Chauhan A Knowledge and attitudes about HIV/AIDS of students in H.P. Government Dental College and Hospital, Shimla, India J Dent Educ 2013 77 09 1218 1224 24002861

[20] Aggarwal A Panat S R Knowledge, attitude, and behavior in managing patients with HIV/AIDS among a group of Indian dental students J Dent Educ 2013 77 09 1209 1217 24002860

[21] Beaton D E Bombardier C Guillemin F Ferraz M B Guidelines for the process of cross-cultural adaptation of self-report measures Spine 2000 25 24 3186 3191 11124735 10.1097/00007632-200012150-00014

[22] Gjersing L Caplehorn J R Clausen T Cross-cultural adaptation of research instruments: language, setting, time and statistical considerations BMC Med Res Methodol 2010 10 10 13 20144247 10.1186/1471-2288-10-13 PMC2831007

[23] Ranauta A Tappuni A R Coulthard P HIV teaching: a dental curriculum which fosters knowledge and attitude Oral Dis 2020 26 01 123 126 32862521 10.1111/odi.13401

[24] Askarian M Mirzaei K Assadian O Iranians' attitudes about possible human immunodeficiency virus transmission in dental settings Infect Control Hosp Epidemiol 2007 28 02 234 237 17265412 10.1086/509860

[25] Madhan B Gayathri H Garhnayak L Naik E S Dental students' regard for patients from often-stigmatized populations: findings from an Indian dental school J Dent Educ 2012 76 02 210 217 22319086

[26] Fauk N K Ward P R Hawke K Mwanri L HIV stigma and discrimination: perspectives and personal experiences of healthcare providers in Yogyakarta and Belu, Indonesia Front Med (Lausanne) 2021 8 625787 34055824 10.3389/fmed.2021.625787 PMC8149745

[27] Szaflarski M Spirituality and religion among HIV-infected individuals Curr HIV/AIDS Rep 2013 10 04 324 332 23996649 10.1007/s11904-013-0175-7 PMC5302191

[28] Arheiam A El Tantawi M Al-Ansari A Arab dentists' refusal to treat HIV positive patients: a survey of recently graduated dentists from three Arab dental schools Acta Odontol Scand 2017 75 05 355 360 28431481 10.1080/00016357.2017.1316867

[29] Ellepola A NB Joseph B K Sundaram D B Sharma P N Knowledge and attitudes towards HIV/AIDS amongst Kuwait University dental students Eur J Dent Educ 2011 15 03 165 171 21762321 10.1111/j.1600-0579.2010.00652.x

[30] McCarthy G M MacDonald J K Gender differences in characteristics, infection control practices, knowledge and attitudes related to HIV among Ontario dentists Community Dent Oral Epidemiol 1996 24 06 412 415 9007360 10.1111/j.1600-0528.1996.tb00890.x

[31] Oliveira E R Narendran S Falcão A Brazilian dental students' knowledge and attitudes towards HIV infection AIDS Care 2002 14 04 569 576 12204159 10.1080/09540120208629675

[32] Taiwo O Dental practice, human immunodeficiency virus transmission and occupational risks: views from a teaching hospital in Nigeria Ann Med Health Sci Res 2014 4 02 S94 S98 25184095 10.4103/2141-9248.138020 PMC4145525

[33] Varas-Díaz N Rivera M Rivera-Segarra E Beyond negative attitudes: Examining HIV/AIDS stigma behaviors in clinical encounters AIDS Care 2017 29 11 1437 1441 28464694 10.1080/09540121.2017.1322679 PMC6053265

[34] McLean A T Wheeler E K Cameron S Baker D HIV and dentistry in Australia: clinical and legal issues impacting on dental care Aust Dent J 2012 57 03 256 270 22924347 10.1111/j.1834-7819.2012.01715.x

[35] Centers for Disease Control and Prevention [Internet] Summary of Infection Prevention Practices in Dental Settings[cited 2021 Sept 6]. Accessed November 4, 2021 at:https://www.cdc.gov/oralhealth/infectioncontrol/summary-infection-prevention-practices/standard-precautions.html

[36] Volgenant C MC Persoon I F de Ruijter R AG de Soet J JH Infection control in dental health care during and after the SARS-CoV-2 outbreak Oral Dis 2021 27 03 674 683 32391651 10.1111/odi.13408 PMC7272817

[37] Farook F F Mohamed Nuzaim M N Taha Ababneh K Alshammari A Alkadi L COVID-19 Pandemic: oral health challenges and recommendations Eur J Dent 2020 14(S 01):S165 S170 33233004 10.1055/s-0040-1718641 PMC7775230

[38] Baytner-Zamir R Lorber M Hermoni D Assessment of the knowledge and attitudes regarding HIV/AIDS among pre-clinical medical students in Israel BMC Res Notes 2014 7 168 24650351 10.1186/1756-0500-7-168 PMC3998113

